# Electronic and Optical Properties of Rocksalt Mg_1−x_Zn_x_O and Wurtzite Zn_1−x_Mg_x_O with Varied Concentrations of Magnesium and Zinc

**DOI:** 10.3390/ma15217689

**Published:** 2022-11-01

**Authors:** Yin-Pai Lin, Sergei Piskunov, Laima Trinkler, Mitch Ming-Chi Chou, Liuwen Chang

**Affiliations:** 1 Institute of Solid State Physics, University of Latvia, 8 Kengaraga str., LV-1063 Riga, Latvia; 2 Center of Crystal Research, Department of Materials and Optoelectronic Science, National Sun Yat-Sen University, 70 Lienhai Rd., Kaohsiung 80424, Taiwan

**Keywords:** rocksalt Mg_1−x_Zn_x_O, wurtzite Zn_1−x_Mg_x_O, electronic structures, optical properties

## Abstract

The structural, electronic and optical properties of rocksalt Mg${}_{1-x}$Zn${}_{x}$O and wurtzite Zn${}_{1-x}$Mg${}_{x}$O with the concentration of Zn and Mg varying from 0.125 to 0.875 were investigated using density functional theory (DFT), DFT+U, linear response theory and the Bethe–Salpeter equation. According to the experimental band gap for varied concentrations of magnesium and zinc, modeling the supercell was utilized for the varied concentrations of Mg/Zn/O compounds in order to not only avoid constructing the complicated interface systems that are observed in the experiments but also take into account the excitonic effects that usually require huge computational resources. From the calculated density of states, the Zn states are highly related to the edge of the conduction band minimum and responsible for the width of bandgap. In addition, the contribution of Zn–*d* states is below expectations as they are located away from the VBM. As for the optical response, an increase in Zn concentration would cause a red-shifted spectrum, on the whole. In contrast, the higher concentration of Mg also triggers the blue-shift of the optical spectrum. In addition, anisotropic properties could be found in the spectrum with consideration of the excitonic effects, whereas there is no apparent difference in optical response based on linear response theory. In addition, the optical features of this work reflect the characteristic peaks of the literature around the absorption onset.

## 1. Introduction

Taking into account their wide optical spectrum from the deep-blue to deep-ultraviolet regions, the compounds or alloys made of MgO and ZnO (ZMO) have been investigated for numerous applications, including light-emitting diode [1], photosensors [2], photocatalysts [3], photodetectors [4], solar cells [5] and thermoelectric devices [6]. Controlling the ratio between the Mg and Zn can realize bandgap engineering from 3.3 to 7.8 eV [7,8,9,10], making ZMO a potential material for many optoelectronic devices. Due to the discrepancy between the crystallographic phases of MgO and ZnO, there are two main crystallographic phases in ZMO, which are rocksalt (RS) and wurtzite (WZ). Both types of ZMO can actually be synthesized for RS Mg${}_{1-x}$Zn${}_{x}$O with x = 0.22–0.87 and WZ Zn${}_{1-x}$Mg${}_{x}$O with x = 0.26–0.66 [7]. In general, optical absorption and photoluminescence measurements are usually applied to analyze the optical response [7,10]. Both MgO and ZnO often contain point defects, which significantly determine and affect their functional properties [11,12,13,14]. Therefore, a deeper understanding of the optical responses of RS and WZ ZMO is essential for further measurements and synthesis.

The frequency-dependent dielectric function is one of the parameters necessary for the evaluation of optical properties including absorption [15], reflectivity and electron energy loss function [16], which can be utilized to compare the results of spectroscopic ellipsometry and optical absorption from the experiments [7]. Due to the recent developments in density functional theory (DFT) approaches, the dielectric function can be easily calculated from the electronic structure. One of the common methodologies is linear response theory [16,17]. However, the precision of DFT within linear response dielectric functions could be under question for the semiconductors and insulators due to the lack of taking into account the electron–hole interactions [17,18,19]. Therefore, the dielectric function of ZnO [20] and MgO [8] within linear response theory would be inaccurately predicted in comparison with experiments even using the GW method of the many-particle perturbation theory to modify the DFT band structures [8]. To describe the excitonic effects, the Bethe-Salpeter equation will be further adopted to calculate the optical spectrum, especially emphasizing the interband transitions around the band gap. Although the models for the optical properties of the ZMO RS and WZ phases have been discussed with different geometries for varied concentrations of doped atoms [8,9], there are few studies that simultaneously exhibit the discrepancy between these two phases with the identical concentration of substituted atoms. It is especially interesting to find out whether the optical response is similar in the cases of RS ZMO and WZ ZMO models with the same ZnO and MgO ratio.

With respect to real systems [21,22], the ZMO composites demonstrate distinct interface phenomena, which reveal the seldom formation of perfect crystal structures without an inevitable point defects vacancy for ZMO alloys, such as vacancy oxygen, interstitial zinc, vacancy zinc and interstitial oxygen. From the theoretical calculations using the concepts of a supercell [8,9], the ZMO models can not only be well-expressed for electronic structures and optical peaks, but are also reasonable for atomic numbers. In contrast, building interface structures with varied concentrations means huge numbers of atomic numbers, which cause a tremendous cost of computational resources, especially for calculating the electron–hole interactions for ZnO and MgO [23]. Although interface structures are important to realize real MgO–ZnO composite systems, the main task of the current study is focusing on the optical response inclusive of the absorption spectrum and transition contribution arising from the varied concentrations for the RS ZMO and WZ ZMO models.

In this work, the structural, electronic, and optical properties were calculated with respect to the RS and WZ ZMO models to investigate the photoabsorption spectrum with varied concentrations of ZMO. The structure of this work is arranged as follows: Section 2 describes the computational method and the details of the ZnO (or MgO) models used in this study, while Section 3 presents the calculated results and discussions including the structural properties, band gap, electronic structures, and optical absorption for all ZMO models under study. Finally, conclusions based on the calculated results are presented in Section 4.

## 2. Computational Methods and Models

**Figure 1:**
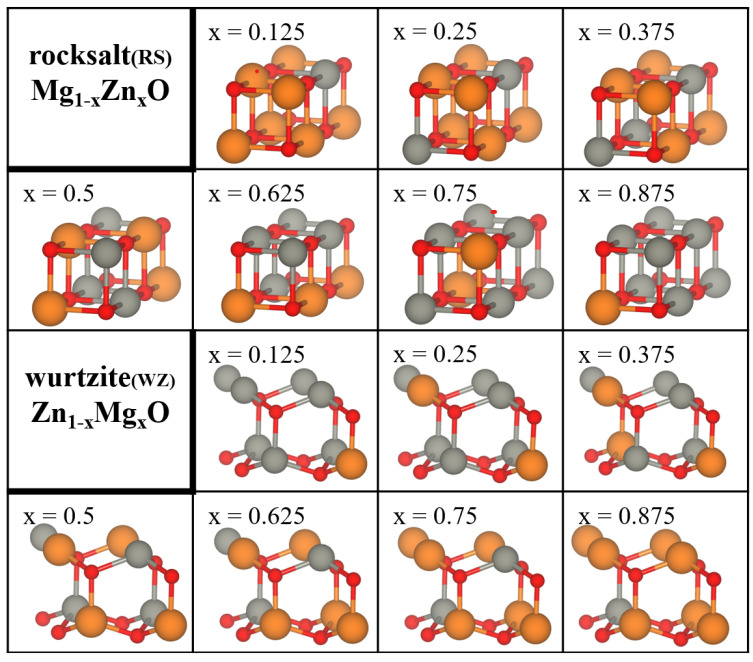
Supercells of 2 × 1 × 1 rocksalt Mg${}_{1-x}$Zn${}_{x}$O and 2 × 2 × 1 wurtzite Zn${}_{1-x}$Mg${}_{x}$O. The concentration (x) of substituted atoms for each of the two ZMO models increases from 0.125 to 0.875. Orange, red and gray spheres represent Mg, O and Zn atoms, respectively.

Models of two phases of ZMO were constructed using the CRYSTAL17 code [24]. The RS oxides model is based on the pure MgO geometry of octahedral coordination; the WZ oxides model is based on hexagonal structure owing to characteristics of the pure ZnO crystal. To control the equivalent concentration, the supercell (SC) 2 × 1 × 1 for RS Mg${}_{1-x}$Zn${}_{x}$O and the SC 2 × 2 × 1 for WZ Zn${}_{1-x}$Mg${}_{x}$O, respectively, were adopted for approaching the ZMO models including 8 oxygen atoms and 8 atoms of magnesium and zinc, as shown in Figure 1. According to the literature, the discrepancy is minor between the electronic and optical properties for the same concentration of ZMO models with different configurations of doped atoms. In order to reduce the possible combinations of ZMO models to save computational cost, one special configuration was utilized for varied concentrations with the doped atoms, which were homogeneously located in the ZMO models [9]. To ensure the reliable calculations of geometric structures, the reciprocal space integrations were obtained by sampling the SCs Brillouin zone with a 8 × 8 × 8 Pack–Monkhorst mesh, which provides 65 k-points in total. The hybrid functional of PBE0 with 25% Hartree–Fock and 75% PBE exchange was applied for the structure optimization [25,26] by means of Broyden–Fletcher–Goldfarb–Shanno (BFGS) algorithm. When the variations in total energy were less than 10${}^{-10}$ a.u. between two successive self–consistent field (SCF) procedures, the calculations were considered to be converged. Meanwhile, the tolerances of 10${}^{-7}$ were set for summations of Coulomb and exchange integrals.

Using the equilibrium lattice parameters of two ZMO phases obtained from CRYSTAL-PBE0, the electronic structures and optical properties were calculated by the atomic simulation environment (ASE) [27,28] and the DFT code GPAW [29,30] based on the projector–augmented wave method. The electronic configurations of valence electrons per atom were as follows: Mg(2s${}^{2}$2p${}^{6}$3s${}^{2}$), O(2s${}^{2}$2p${}^{4}$) and Zn(3d${}^{10}$4s${}^{2}$). For the structural optimization, the geometric constitutions were relaxed until the maximum force of atoms was below 0.05 eV/Å within the same lattice parameters of CRYSTAL-PBE0 according to BFGS algorithm. In Supplementary S1 and S2, the influence on the structural models of CRYSTAL and GPAW are discussed in detail. Monkhorst–Pack k–point grids with 8 × 8 × 8 were used for Brillouin zone sampling during the relaxations and SCF procedures. In consideration of the balance between the computational cost and accuracy, the PBE and the Gritsenko–van Leeuwen–van Lenthe–Baerends functional with the solid-state modification (GLLBSC) [31] functionals were chosen to perform the electronic structure calculations. For the purpose of overcoming the underestimated band gap energy ($E_{g}$) of ZMO models, the Hubbard DFT+U term [32] was utilized to interpret the localized phenomena of electrons. The Hubbard correction of U${}_{p,Mg}$ and U${}_{d,Zn}$ were 0.7 [33] and 10 [20] eV, respectively. The energy cutoff for the plane-wave (PW) basis expansion was chosen to be 500 eV. The convergence criteria were the same as the default.

In the calculations of optical properties of all ZMO models, the linear density response function (LR) on the basis of projector-augmented wave method [17] and the Bethe–Salpeter equation (BSE) [17,18,19] for excitonic effects were applied to predict the frequency-dependent dielectric function ϵ(ω), including the real $\epsilon_{Re}$(ω) and imaginary $\epsilon_{Im}$(ω) part of ϵ(ω). For the LR ϵ(ω), random phase approximation (RPA) was adopted and the PW energy cutoff to determine the dielectric matrix was determined at 100 eV. For the BSE ϵ(ω), five valence and five conduction bands were obtained to ensure the convergence of the optical spectrum [8] as well as the PW energy cutoff to obtain the dielectric matrix, which was determined at 50 eV. The absorption coefficient ($\alpha_{abs}$) was transformed using the following relation [15]:

$$\alpha_{abs}=\sqrt{2}\omega {\left(\sqrt{\epsilon_{Re}^{2}\left(\omega \right)+\epsilon_{Im}^{2}\left(\omega \right)}-\epsilon_{Re}\left(\omega \right)\right)}^{1/2}.$$

VESTA software [34] was used to express the atomic environments; Python packages of NumPy [35] and Matplotlib [36] were adopted to analyze data and produce figures.

## 3. Results and Discussions

### 3.1. Structural Properties and Band Gap

All the equilibrium lattice parameters with different concentrations of substituted atoms for the ZMO models of RS and WZ phases are listed in Table 1. To start with, the experimental lattice constants of RS MgO bulk are around 4.2 Å [37]. In contrast, the theoretical predictions of the optimized lattice constants of RS MgO bulk are in the range between 4.16 Åand 4.32 Åfollowing DFT calculations. Although the RS ZMO models with different concentrations of doped zinc atoms would slightly change the orthogonal shape of the angles for RS phases, the lattice parameters are in overall agreement with those reported in the literature for RS phases [37,38]. From the experiments performed for WZ ZnO, the lattice constants are 3.250 Å and 5.211 Å for the a and c axis, respectively [37]. For the WZ ZMO models, the structures sustained the properties of the WZ phases of ZnO. In brief, the varied lattice constants of the WZ phases are more sensitive to concentration than that for the RS phases depending on concentration. Both the RS and WZ ZMO models predict the increasing lattice constants with the denser concentration for lattice constants in the a and b axis. However, the WZ ZMO models indicate that the denser concentration of doped magnesium atoms would reduce the lattice constants in the c axis. Indeed, it was confirmed that the increasing concentration of substituted atoms causes the phase transitions of the crystallographic between the RS and WZ phases [39]. The crystallographic geometry has a direct influence on electronic structures and optical properties, but investigation of phase transitions is beyond the scope of the current study.

**Figure 2:**
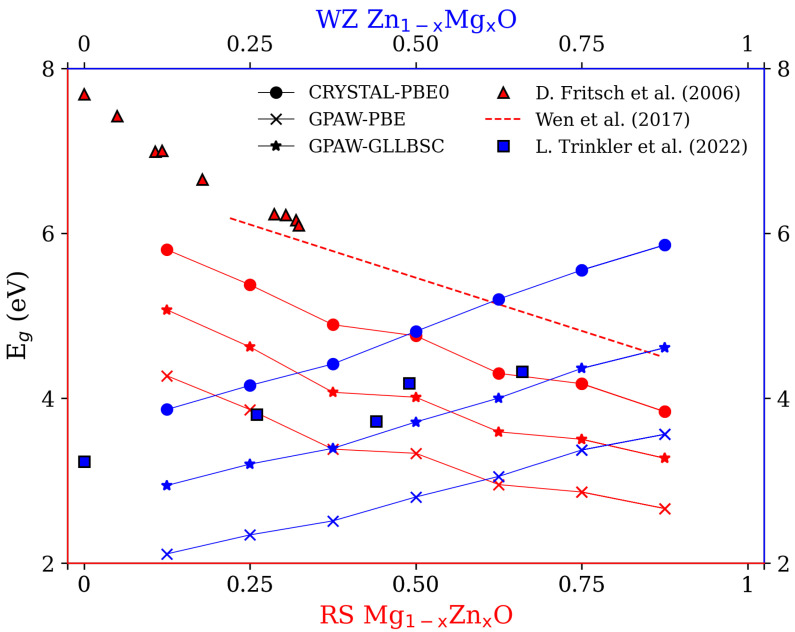
Band gaps ($E_{g}$) for rocksalt (RS) Mg${}_{1-x}$Zn${}_{x}$O and wurtzite (WZ) Zn${}_{1-x}$Mg${}_{x}$O for x = 0.125, 0.25, 0.375, 0.5, 0.625, 0.75 and 0.875. The blue color is WZ phase; the red color is RS phase. The symbols of circles, crosses and stars represent the $E_{g}$ calculated by PBE0 with CRYSTAL17, PBE+U with GPAW and GLLBSC+U with GPAW, respectively. The red triangles and blue squares stand for the experimental values for RS ZMO alloys [39] and WZ ZMO epilayers [7]. The red dashed line is the fitting relation between $E_{g}$ and concentrations of RS Mg${}_{1-x}$Zn${}_{x}$O ranging from x = 0.22 to 0.87 based on the experimental values of $E_{g}$ (RS Mg${}_{1-x}$Zn${}_{x}$O) = 4.17 + 2.58(1−x) (eV) [10].

The experimental and theoretical band gaps ($E_{g}$) calculated from the hybrid functional within CRYSTAL17 and DFT+U within GPAW are shown in Figure 2. From the literature [7,10,39,40,41,42,43,44], the optical spectrophotometer systems are commonly obtained to determine the $E_{g}$ for the fabricated ZMO samples. In this work, the $E_{g}$ of RS Mg${}_{1-x}$Zn${}_{x}$O and WZ Zn${}_{1-x}$Mg${}_{x}$O with varied concentrations are from 4.5 up to 7.8 eV (red triangles, red dashed line) [10,39] and from 3.2 to 4.4 eV (blue squares) [7], respectively. The larger percentage of Zn atoms the structures have, the narrower the $E_{g}$ is. Roughly, the theoretical variations in $E_{g}$ for RS and WZ ZMO models satisfy the trends of experiments in Figure 2. Nevertheless, there is still not very good agreement for the $E_{g}$ in comparison with the experiments. On one hand, the PBE0 including 25% Hartree–Fock would overestimate the $E_{g}$ owing to the larger fractions of exact exchange, whereas the very large $E_{g}$ of the insulator would be underestimated by PBE0 functionals [45]. The $E_{g}$ of ZMO models with CRYSTAL-PBE0 are in agreement with this phenomena. On another hand, the $E_{g}$ calculated by means of GPAW-PBE and GPAW-GLLBSC with DFT+U are smaller than the values obtained by means of CRYSTAL-PBE0. Although both GPAW-PBE and GPAW-GLLBSC greatly underestimate the $E_{g}$ for RS Mg${}_{1-x}$Zn${}_{x}$O models, the GPAW-GLLBSC predict well the WZ $E_{g}$ in comparison with the CRYSTAL-PBE0 and GPAW-PBE. In the light of the computational cost, the GPAW-GLLBSC with DFT+U will be utilized for the calculations in the sections on electronic structures and optical absorption.

### 3.2. Electronic Structures

**Figure 3:**
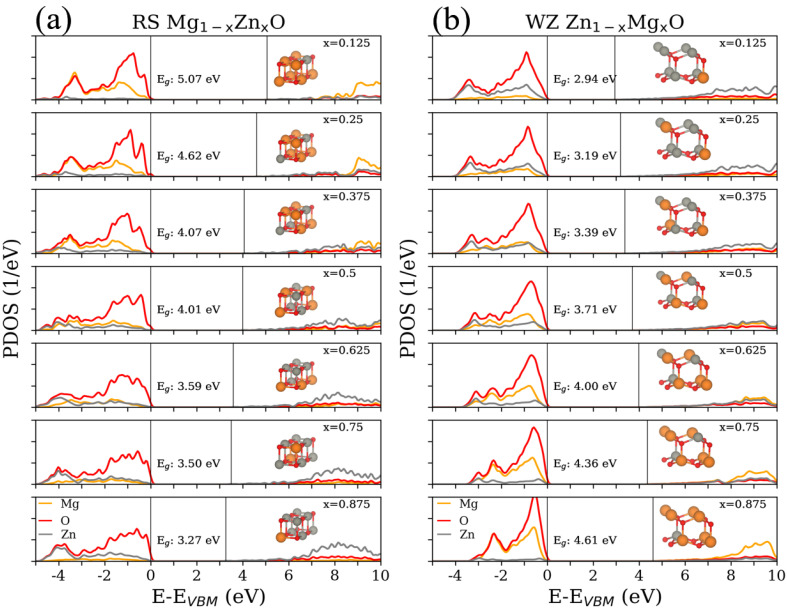
Partial density of states (PDOS) for (**a**) rocksalt (RS) Mg${}_{1-x}$Zn${}_{x}$O and (**b**) wurtzite (WZ) Zn${}_{1-x}$Mg${}_{x}$O at different concentrations (x = 0.125, 0.25, 0.375, 0.5, 0.625, 0.75 and 0.875) with respect to the valence band maximum (VBM). The colors of orange, red and gray are the PDOS of magnesium (Mg), oxygen (O) and zinc (Zn), respectively. One vertical solid line is the VBM at 0 eV, and the other vertical solid line is the conduction band minimum above 0 eV. The insets inside each panel correspond to the models in Figure 1.

The calculated partial density of states (PDOS) for the RS Mg${}_{1-x}$Zn${}_{x}$O and WZ Zn${}_{1-x}$Mg${}_{x}$O are shown in Figure 3. Starting with O atoms, the oxygen always dominates in the states around the valance band maximum (VBM) for the RS and WZ phases with any concentration. For the RS Mg${}_{1-x}$Zn${}_{x}$O, the increasing concentration of Zn causes the broader distributions of oxygen valence bands and reduces the obvious peak around –1 eV (x = 0.125 to 0.375). In contrast, the increasing concentration of Mg triggers the narrower distributions of oxygen valence bands from the range of 4 to 3 eV for the case of WZ Zn${}_{1-x}$Mg${}_{x}$O. In addition, the denser concentration of Mg also lets the PDOS of oxygen, first peaking at around –1 eV, become larger and slightly shift to VBM. Furthermore, the functions of Mg and Zn with the varied concentrations appear to be somewhat different for the RS and WZ phases. In the valence band regions of RS Mg${}_{1-x}$Zn${}_{x}$O, there are only two main peaks at around –1.5 eV and –3.5 eV for the initial x = 0.125. With the increasing concentration of Zn, the deeper peaks shifted to –4 eV, as well as there being no obvious peaks at around –1.5 eV. For the WZ Zn${}_{1-x}$Mg${}_{x}$O, there are also two main peaks at around –1 eV and –3.5 eV. Unlike the RS ZMO models, the increasing concentration of Mg could cause the peaks around VBM to become more distinct and shift the deeper peak to higher energy, at around –2.5 eV. Consequently, the states of Mg tend to interact with the O states around the VBM. As for the conduction band regions, the increasing concentration of Zn forms a series of states which are related to decrease in the $E_{g}$. A larger amount of Mg atoms would create conduction bands at higher energy.

**Figure 4:**
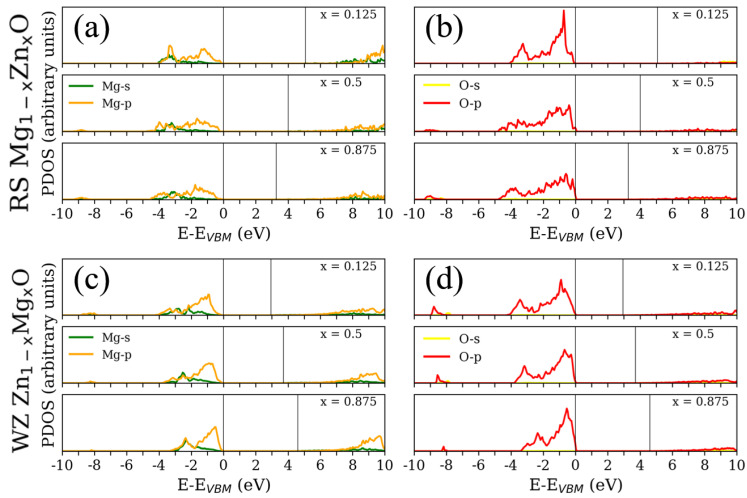
Partial density of states (PDOS) for (**a**,**b**) rocksalt (RS) Mg${}_{1-x}$Zn${}_{x}$O and (**c**,**d**) wurtzite (WZ) Zn${}_{1-x}$Mg${}_{x}$O at different concentrations (x = 0.125, 0.5 and 0.875) with respect to the valence band maximum (VBM). The colors of green, orange, yellow and red correspond to the Mg–*s*, Mg–*p*, O–*s* and O–*p* states, respectively. One vertical solid line is the VBM at 0 eV, and the other vertical solid line is the conduction band minimum above 0 eV.

**Figure 5:**
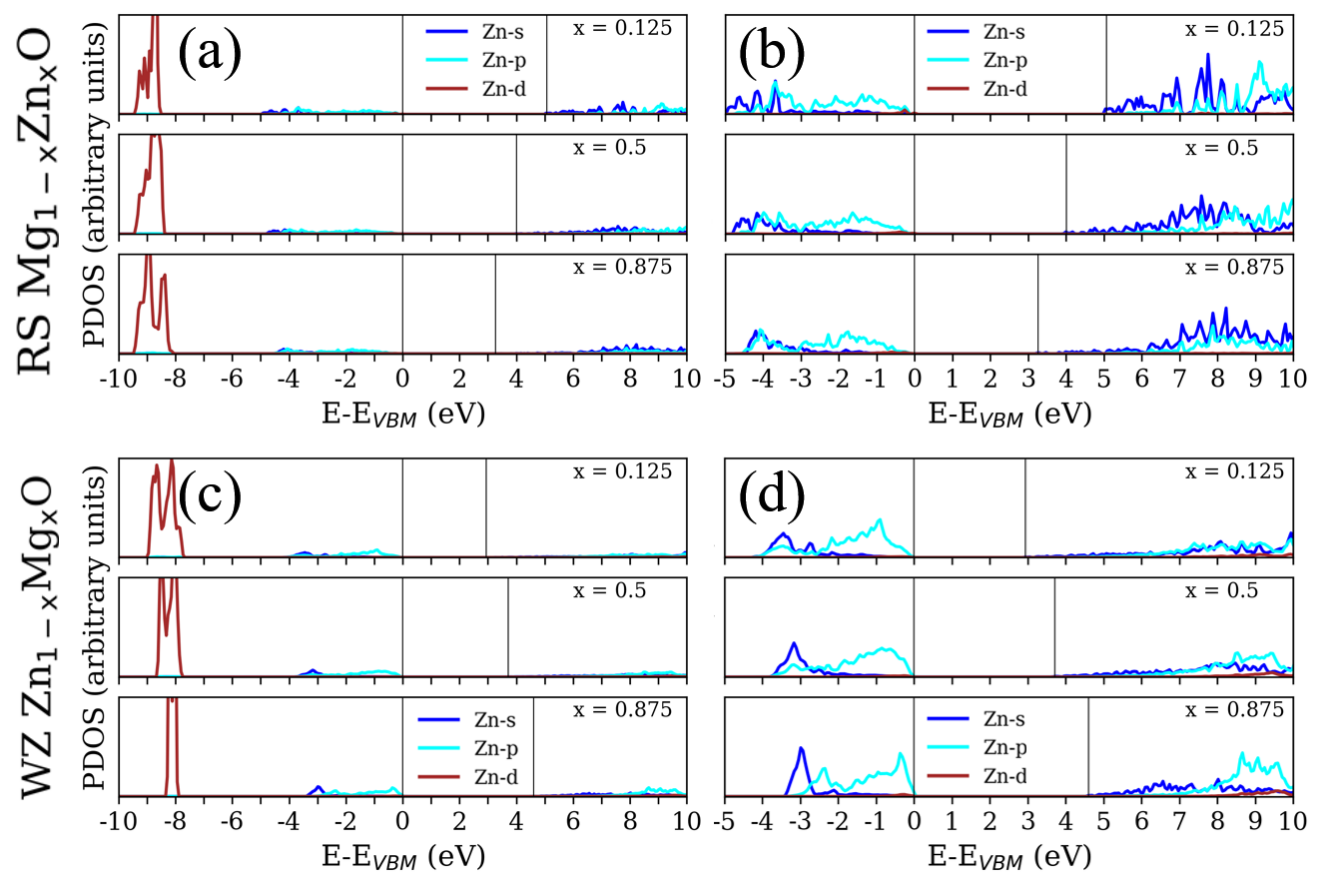
Partial density of states (PDOS) for (**a**,**b**) rocksalt (RS) Mg${}_{1-x}$Zn${}_{x}$O and (**c**,**d**) wurtzite (WZ) Zn${}_{1-x}$Mg${}_{x}$O at different concentrations (x = 0.125, 0.5 and 0.875) with respect to the valence band maximum (VBM). The colors of blue, cyan and brown correspond to the Zn–*s*, Zn–*p* and Zn–*d* states, respectively. The PDOS of (**a**,**c**) are scaled by a factor of 1/3 in comparison with (**b**,**d**) and Figure 4. One vertical solid line is the VBM at 0 eV, and the other vertical solid line is the conduction band minimum above 0 eV.

To obtain more insight into the detailed states, Figure 4 and Figure 5 illustrate the PDOS on *s*, *p* and *d* states for Mg, O and Zn. To simplify the illustrations, there are only three concentrations (x = 0.125, 0.5 and 0.875) reported in Figure 4 and Figure 5. Near the VBM, the valence bands are mainly composed of O–*p* states for both RS and WZ ZMO models. In addition, the increasing Mg concentration would trigger the O–*p* states to centralize and become closer to VBM. With respect to Mg states, there are no sharp fluctuations regardless of the *s* and *p* states in the valance band regions. In contrast, a decreasing concentration of Mg would be obviously related to the reductions in the *p* state at higher energies in the conduction bands. In brief, the major contributions around VBM are the states of Mg and O. Around the conduction band minimum (CBM), there are no apparent occupied states arising from Mg and O atoms.

From the literature, it is known that strong exchange and correlation interactions arise between hybridised O–*p* and Zn–*d* states for RS ZMO models [46]. Similarly, the DOS consists essentially of Zn–*d* and O–*p* states near the Fermi level for the compounds of WZ ZMO [47]. However, the intensities of Zn–*d* states are not strong enough around the VBM until –5 eV [8]. In addition, the most Zn–*d* states are located at around –7.5 eV and there is a series of zero states from –5 to –7.5 eV [9]. Specifically, it is important to understand the reason for the shifting Zn–*d* states. Indeed, the Hubbard U for correcting the hybridization of Zn and O would cause the 3*d*-band of Zn to shift to the range between –7.5 to –10 eV, which properly describes the electronic structures, as well as being in agreement with the experimentally obtained dielectric function, which was proposed by Calzolari et al. [48]. Therefore, the PDOS based on the DFT+U would modify the locations of the valance bands of Zn–*d* states. Regarding the modified PDOS, the states around VBM are dominated by the strong hybridization among the *p* states of Mg, O and Zn, as well as the *s* states of Zn showing major hybridisation near CBM. A sequence of Zn and O states hybridize with each other around CBM when the ratio of Zn concentration increases. This mechanism fulfills the formation of the bonding and antibonding phenomenon for Zn and O [8,46], which triggers the reduction in $E_{g}$ and CBM. Briefly, both $E_{g}$ and DOS could significantly affect optical response. The GLLBSC with Hubbard correction not only presents a reasonable $E_{g}$ in comparison with the hybrid funtionals and experiments, but also provides the proper valence and conduction band positions.

### 3.3. Optical Absorption

**Figure 6:**
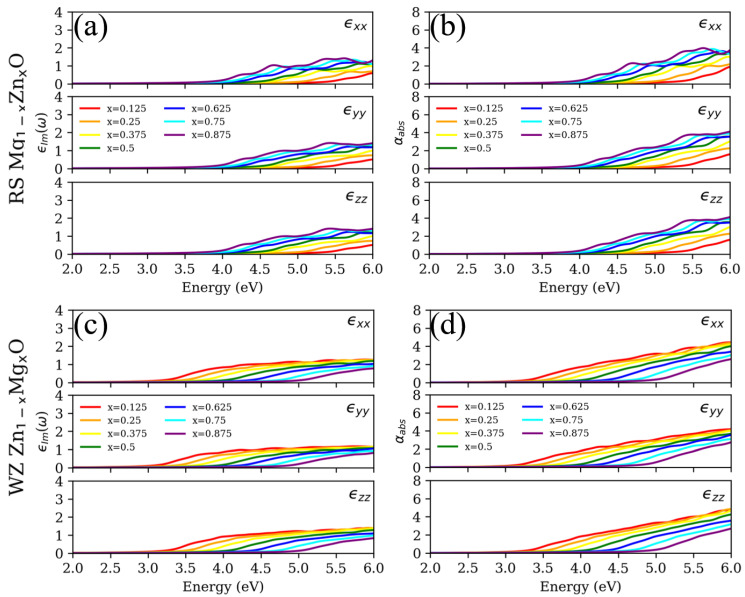
(**a**,**c**) Imaginary part of the dielectric function ($\epsilon_{Im}$) and (**b**,**d**) absorption coefficient ($\alpha_{abs}$) for rocksalt (RS) Mg${}_{1-x}$Zn${}_{x}$O and wurtzite (WZ) Zn${}_{1-x}$Mg${}_{x}$O based on linear response theory within RPA. The first rows (**a**,**b**) demonstrate the RS phases; the second rows (**c**,**d**) represent the WZ phases. The top, middle and bottom panel for each sub-figures correspond to the direction of $\epsilon_{xx}$, $\epsilon_{yy}$ and $\epsilon_{zz}$, respectively. The red, orange, yellow, green, blue, cyan and purple lines individually correspond to the concentrations (x) of 0.125, 0.25, 0.375, 0.5, 0.625, 0.75 and 0.875.

On the basis of the electronic structures obtained in the previous section, Figure 6 shows the calculated LR ϵ(ω) and LR $\alpha_{abs}$ via RPA for all the ZMO models shown in Figure 1. It is known that the larger percentage of MgO atoms ZMO has, the wider the $E_{g}$ is. These circumstances have an influence on the shift in the spectrum depending on the percentage between magnesium and zinc. The red-shifted spectra are observed from the increasing concentration in the RS Mg${}_{1-x}$Zn${}_{x}$O. By contrast, the blue-shifted spectra are predicted as the concentration of MgO increases for WZ Zn${}_{1-x}$Mg${}_{x}$O. According to the results obtained for electronic structures, it can be expected that the calculated absorption spectrum of RS ZMO models would be strongly red shifted compared to the experimental measurements of pure RS MgO [8]. As for the WZ ZMO models, the spectrum would be slightly red shifted according to the experiments [7]. Although the dielectric function meets the trend of the shifted spectrum, there are two main obstacles for the predicted spectrum with LR via RPA. One is the disappearance of excitonic bound states around the $E_{g}$; the other is the vagueness of anisotropic properties. As far as the experimental absorption spectrum is concerned, the excitonic bound states of pure RS MgO and WZ ZnO can be observed at around 7.8 [8] and 3.3 [7] eV, respectively. As to the anisotropic properties, hardly do the SCs of geometric structures with different concentrations remain symmetrical for both RS and WZ ZMO models in each direction. Yet, the spectrum with LR via RPA are almost the same in each direction for the identical crystallographic phases.

**Figure 7:**
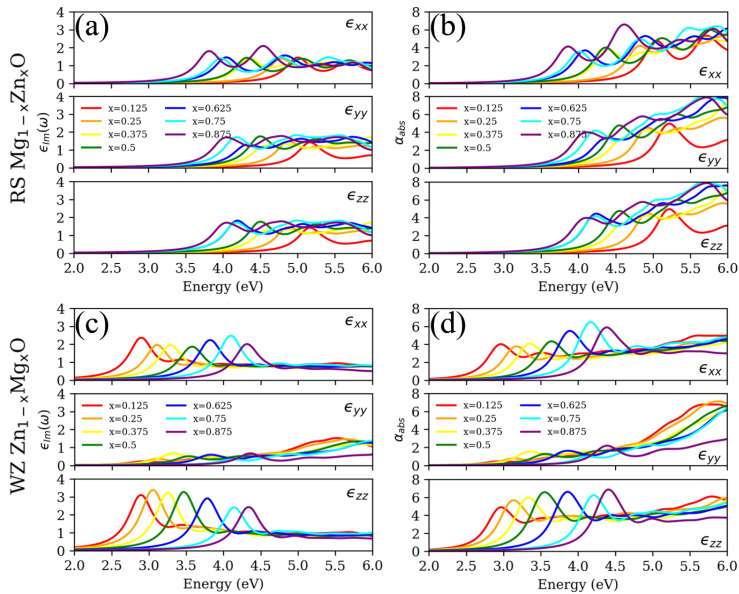
(**a**,**c**) Imaginary part of the dielectric function ($\epsilon_{Im}$) and (**b**,**d**) absorption coefficient ($\alpha_{abs}$) for rocksalt (RS) Mg${}_{1-x}$Zn${}_{x}$O and wurtzite (WZ) Zn${}_{1-x}$Mg${}_{x}$O based on Bethe–Salpeter equation. The symbols, colors and legends are identical to the descriptions in Figure 6.

Considering the excitonic effects, it is absolutely necessary to construct the exact spectral characteristic for both RS MgO and WZ ZnO, including the absorption onset and entire spectral appearance [23]. After taking into account the electron–hole interaction in accordance with BSE, Figure 7 demonstrates the pronounced excitonic peak around the absorption onset, which cannot be observed in Figure 6. For the RS Mg${}_{1-x}$Zn${}_{x}$O, the excitonic peaks are constantly red-shifted with increasing concentrations of Zn. Conversely, the excitonic peaks are gradually blue shifted with increasing concentrations of Mg for the WZ Zn${}_{1-x}$Mg${}_{x}$O. The trends of spectral shift are in agreement with previously calculated electronic structures and $E_{g}$. Meanwhile, the higher concentration of Zn roughly enhances the peak value of BSE $\epsilon_{Im}$(ω) in the x, y and z directions. In Supplementary S3, the spectra based on the modified electronic structures are also presented via the BSE methods, which presents the blue-shifted absorption onset and entire spectrum owing to the improvement in the underestimated $E_{g}$. From the perspectives of anisotropic properties, both $\epsilon_{Im}$(ω) and $\alpha_{abs}$ via BSE reveal anisotropic absorption due to the geometric structure of SC. Particularly, the most distinctive discrepancy of optical spectrum is the WZ Zn${}_{1-x}$Mg${}_{x}$O in the y orientation. The magnitude of excitonic peaks is much smaller than the response in the x and z directions. In addition, the spectral features around the absorption onset are smoothly increasing, not sharply enhancing. From the experiments, the disappearance or vagueness of excitonic peaks is actually observed for the WZ Zn${}_{0.56}$Mg${}_{0.44}$O, Zn${}_{0.51}$Mg${}_{0.49}$O and Zn${}_{0.34}$Mg${}_{0.66}$O [7]. It is emphasized that the true images of experiments for ZMO samples show the complex surface structures, not the uniform lattice structures [21,22]. From the literature [7], the disappearance or vagueness of excitonic peaks may also indicate the composite structures that reflect the superposition of the optical spectra for both RS and WZ phases. Indeed, the interface systems, including doping and defect, could also provide different optical characteristics for the spectrum. However, there are few studies that emphasize that the anisotropic properties may trigger the disappearance or vagueness of excitonic peaks around the absorption onset. In short, neglecting the excitonic effects not only motivates the underestimated contributions of excitons around the $E_{g}$, but also misleads the anisotropic properties arising from the structural factors.

## 4. Conclusions

In summary, the geometry, electronic structures and optical properties for the RS and WZ phases of ZMO models were investigated along with controlled concentrations of Mg and Zn. Essentially, most of the properties of the RS and WZ phases of ZMO compounds are very different from each other. First, the varied concentrations of Zn provide more opportunities for hybridisation with the other elements of ZMO. Although the varied concentrations of Mg also have an influence on the tuning of the $E_{g}$, the bonding formations between Zn and O states directly affect the edge of CBM and, thus, the width of $E_{g}$. The abundant conduction states of Zn around CBM also allow the diversity of interband transition in the optical spectrum. Meanwhile, the effect of Zn–*d* states is not distinct due to the band energy locations away from the VBM. Fundamentally, the transition states around the $E_{g}$ are mainly contributed by the *p* states of O, Mg and Zn to *s* and *p* states of Zn. Therefore, the concentration of Zn is of primary importance, regardless of the RS or WZ phases. Second, the positions of doped atoms would induce the anisotropic properties which trigger a diversity of optical response in the different orientations. The WZ phases of ZMO models are more diverse than RS phases, in general. For the $\epsilon_{yy}$ of WZ Zn${}_{1-x}$Mg${}_{x}$O, the anisotropic properties cause the excitonic bound states to be smaller than for the other directions, and trigger the spectrum without a sharp peak around the absorption onset. Notably, the applications of ZMO models with a reasonable computational cost have a good tendency in comparison with the experiments. However, there is a lack of theoretical evidence for how identical the supercell and interface models are in consideration with the experiments and this is an area which needs to be developed in the future. Finally, the present study may be useful to design and model RS Mg${}_{1-x}$Zn${}_{x}$O and WZ Zn${}_{1-x}$Mg${}_{x}$O for optoelectronic and photocatalytic systems.

## Figures and Tables

**Table 1:** Equilibrium lattice parameters of 2 × 1 × 1 rocksalt (RS) Mg${}_{1-x}$Zn${}_{x}$O and 2 × 2 × 1 wurtzite (WZ) Zn${}_{1-x}$Mg${}_{x}$O at different concentrations of substituted atoms.

x	a (Å)	b (Å)	c (Å)	α (${}^{\circ }$)	β (${}^{\circ }$)	γ (${}^{\circ }$)
	RS, WZ					
0.125	8.429, =a${}_{0}$	4.213, =b${}_{0}$	4.213, 5.196	=$\alpha_{0}$, =$\alpha_{0}$	=$\beta_{0}$, =$\beta_{0}$	=$\gamma_{0}$, =$\gamma_{0}$
0.25	8.453, =a${}_{0}$	4.220, 6.521	4.220, 5.183	=$\alpha_{0}$, =$\alpha_{0}$	89.999, =$\beta_{0}$	=$\gamma_{0}$, 120.005
0.375	8.446, 6.519	4.231, 6.518	4.231, 5.167	=$\alpha_{0}$, 90.024	=$\beta_{0}$, 89.978	=$\gamma_{0}$, 119.860
0.5	8.506, 6.532	4.232, 6.517	4.232, 5.148	=$\alpha_{0}$, 89.951	89.995, 90.025	=$\gamma_{0}$, 119.925
0.625	8.502, 6.529	4.248, 6.529	4.248, 5.125	=$\alpha_{0}$, 89.980	89.999, 90.020	=$\gamma_{0}$, 119.844
0.75	8.527, 6.542	4.250, 6.542	4.253, 5.102	=$\alpha_{0}$, =$\alpha_{0}$	89.885, =$\beta_{0}$	=$\gamma_{0}$, 119.997
0.875	8.533, 6.554	4.258, 6.554	4.264, 5.074	=$\alpha_{0}$, 89.999	89.946, 90.001	=$\gamma_{0}$, 119.998

RS: a_0_ = 8.408, b_0_ = 4.204, c_0_ = 4.204, α_0_ = 90, β_0_ = 90 and γ_0_ = 90; WZ: a_0_ = 6.520, b_0_ = 6.520, c_0_ = 5.211, α_0_ = 90, β_0_ = 90 and γ_0_ = 120.

## Data Availability

The raw/processed data required to reproduce these findings cannot be shared at this time as the data also form a part of an ongoing study.

## Supplementary Materials

The following supporting information can be downloaded at: https://www.mdpi.com/article/10.3390/ma15217689/s1, S1: Structural models obtained from CRYSTAL and GPAW (text, 1 Table and 1 Figure); S2: Band gaps for the different structural models obtained from CRYSTAL and GPAW (text and 1 Figure); S3: Optical properties of rocksalt ZMO with modified electronic structures (text and 1 Figure). Reference [49] are cited in the Supplementary Materials.
