# Supplementary material for: Electronic and Optical Properties of Rocksalt Mg1−xZnxO and Wurtzite Zn1−xMgxO with Varied Concentrations of Magnesium and Zinc
Source: Materials (Basel). 2022 Nov 1;15(21):7689. doi: 10.3390/ma15217689 (PMC9653927; doi:10.3390/ma15217689)
Supplement: Supplementary file 1 [file materials-15-07689-s001.zip › materials-1970692-supplementary.pdf]

Article

Supplementary information for "Electronic and optical properties of rocksalt $Mg_{1-x}Zn_xO$ and wurtzite $Zn_{1-x}Mg_xO$ with varied concentration of magnesium and zinc"

Yin-Pai Lin[1], Sergei Piskunov[1,*], Laima Trinkler[1], Mitch Ming-Chi Chou[2], LiuWen Chang[2]

1 Institute of Solid State Physics, University of Latvia, 8 Kengaraga str., Riga LV-1063, Latvia
2 Center of Crystal Research, Department of Materials and Optoelectronic Science, National Sun Yat-Sen University, 70 Lienhai Rd., Kaohsiung 80424, Taiwan,

* Correspondence: piskunov@cfi.lu.lv

S1. Structural models obtained from CRYSTAL and GPAW

The strategy of constructing the ZMO models with varied concentration is based on the equilibrium lattice constants calculated by CRYSTAL. Without constraining the lattice constants during the optimization, the main discrepancy of optimized RS ZMO models between CRYSTAL and GPAW is in lattice constants as it is shown in Table S1. The optimized structures within Hubbard U parameters would cause the decreasing lattice constants for the phases of rocksalt and wurtzite in comparison with the experiments and theoretical calculations without Hubbard U parameters proposed by Ma et al [1]. The optimized structures of RS ZMO models calculated by CRYSTAL and GPAW are still consistent with the phase of rocksalt. Both crystal structures of RS ZMO models with and without constraints are congruous with the rocksalt phase shown in Figure 1.

Table S1. Equilibrium lattice constant of 2 × 1 × 1 rocksalt $Mg_{1-x}Zn_xO$ for CRYSTAL and GPAW with varied concentration.

x	a (Å) CRYSTAL,GPAW	b (Å)	c (Å)	α (°)	β (°)	γ (°)
0.125	8.429, 7.768	4.213, 3.883	4.213, 3.883	$= \alpha_0$, 89.999	$= \beta_0$, 89.999	$= \gamma_0$, 89.999
0.25	8.453, 7.797	4.220, 3.896	4.220, 3.896	$= \alpha_0$, 89.999	89.999, $= \beta_0$	$= \gamma_0$, $= \gamma_0$
0.375	8.446, 7.835	4.231, 3.918	4.231, 3.918	$= \alpha_0$, 89.999	$= \beta_0$, 89.999	$= \gamma_0$, 89.999
0.5	8.506, 7.868	4.232, 3.928	4.232, 3.928	$= \alpha_0$, 89.999	89.995, 89.999	$= \gamma_0$, 89.999
0.625	8.502, 7.897	4.248, 3.948	4.248, 3.948	$= \alpha_0$, $= \alpha_0$	89.999, 89.999	$= \gamma_0$, 89.999
0.75	8.527, 7.926	4.250, 3.962	4.253, 3.962	$= \alpha_0$, 89.999	89.885, $= \beta_0$	$= \gamma_0$, $= \gamma_0$
0.875	8.533, 7.959	4.258, 3.979	4.264, 3.979	$= \alpha_0$, 89.999	89.946, 89.999	$= \gamma_0$, 89.999

RS : $a_0 = 8.408$, $b_0 = 4.204$, $c_0 = 4.204$, $\alpha_0 = 90$, $\beta_0 = 90$ and $\gamma_0 = 90$;
CRYSTAL : The lattice constants of RS ZMO models are the same as the values in Table 1.

In contrast to the optimized RS models, the WZ models without any constraints are tend to trigger the phase transition during the optimization calculated by GPAW. In Figure S1, the optimized structures of WZ models by GPAW are shown in comparison with the results obtained by CRYSTAL. Without any constraints during optimization, the equilibrium geometries of WZ ZMO are tend to exhibit the phase of rocksalt. Once the constraints of lattice constants are applied to the optimization, the structures of WZ ZMO models could be still in agreement with the phases of wurtzite. Although the restrictions of optimized settings would cause the imprecise predictions for the electronic structures, the tendency is still in agreement with the experimental values of E_g in Figure 2.

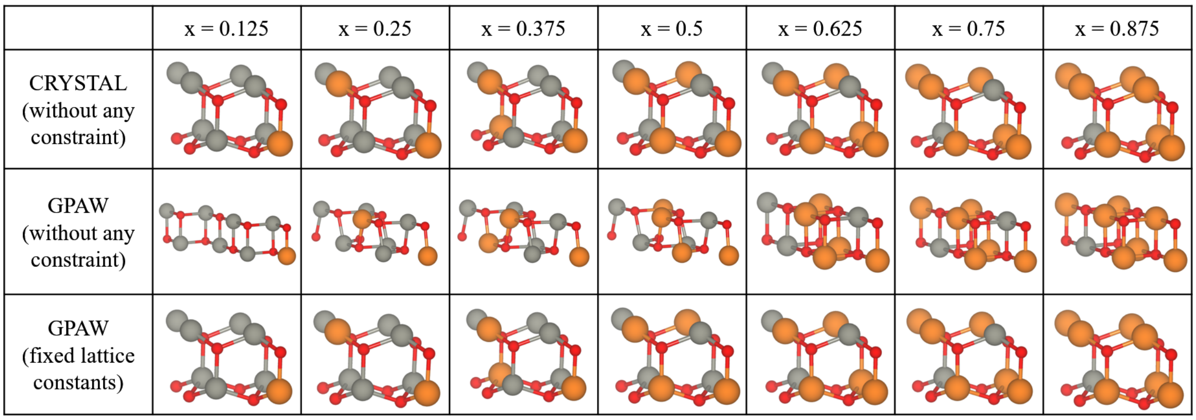

Figure S1. Crystal structures of wurtzite $Zn_{1-x}Mg_xO$ models. The first row is the optimized structures by CRYSTAL. For the equilibrium structures by GPAW, the second and third rows correspond to the fully optimized and constrained lattice constants, respectively. The lattice parameters of third row are obtained from the calculations of first row.

S2. Band gaps for the different structural models obtained from CRYSTAL and GPAW

In the previous section, the difference of optimized structures with and without constraint are discussed. It is also necessary to understand the effect on the E_g. In Figure 2, comparisons of E_g show that the optimized structures calculated by CRYSTAL without constraints and by GPAW with fixed lattice constants are in agreement with the tendency observed in experiments, except the accuracy. Therefore, the E_g of each models in section S1 are further considered to evaluate the precision of the band gap. To simplify the illustrations, the GPAW-GLLBSC with Hubbard correction U are adopted in comparison with CRYSTAL-PBE0 and experiments. The full optimization and DFT+U are beneficent to improve the E_g of RS ZMO models. However, the full optimization would induce the decreasing lattice constants of RS ZMO models in Table S1. However, not only the tendency of E_g but also the precision are performed well in comparison with the experiments with varied concentration for RS ZMO. In contrast, the WZ ZMO models demonstrate the distinct deviation of the E_g with varied concentration. First, the wider E_g is predicted for the full optimization of WZ ZMO models with x = 0.125. From the literature [1] it is known that the E_g of pure RS ZnO are 0.84, 4.05 and 2.45 eV for PAW-PBE, GGA+U_d+U_p^f and experiment, respectively. Meanwhile, the ZMO with the concentration of x = 0.625, 0.75 and 0.875 also present the wider E_g than the structures calculated by means of CRYSTAL-PBE0 and GPAW-GLLBSC with constraint. In consideration of the crystal structures in the second row of Figure S2, the optizmied structures of WZ ZMO with x = 0.125, 0.625, 0.75 and 0.875 are much closer to the phase of rocaksalt. On the other hand, the optimized structures of WZ ZMO with x = 0.25, 0.375 and 0.5 reveal the properties of wurtzite. In the second row of Figure S2, the angles ∠O–Zn–O of x = 0.25, 0.375 and 0.5 are not so orthogonal in comparison with the cases of x = 0.125, 0.625, 0.75 and 0.875. Due to this features, the E_g of x = 0.25, 0.375 and 0.5 are more aligned with the results of WZ GPAW-GLLBSC with constraint. Nevertheless, the lattice geometries are similar to the rocksalt for x = 0.25, 0.375 and 0.5.

In a summary, the full optimizations of ZMO models for rocksalt show an excellent prediction of E_g. Conversely, the optimized geometries without constraint for wurtzite would induce the phase transition during the relaxations. In order to control the phases for both rocksalt and wurtzite during the calculations, the ZMO models are based on the optimized structures calculated by CRYSTAL to undergo the GPAW calculations for electronic structures and optical properties in this work.

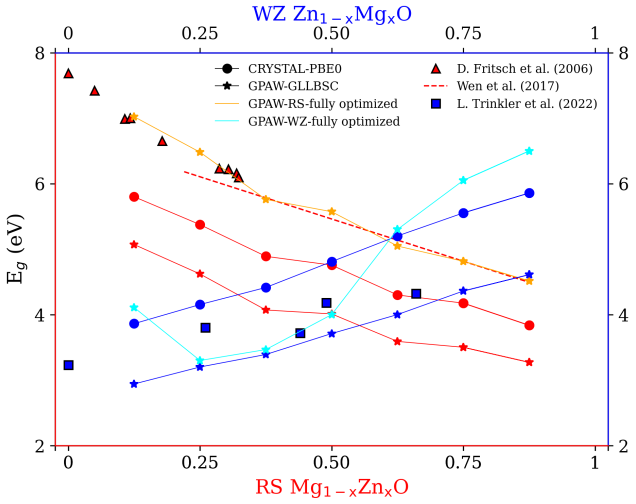

Figure S2. Dependence of band gap (E_g) *vs.* varied concentration for rocksalt (RS) and wurtzite (WZ) ZMO. The curves in orange colors represent the RS ZMO models in Table S1. The curves in cyan colors correspond to the WZ models without any constraint in the second row of Figure S1. The rest of the colors and symbols are identical to the definitions in Figure 2.

S3. Optical properties of rocksalt ZMO with modified electronic structures

Due to the underestimation of RS E_g in Figure 2, the absorption onset and entire spectrum are obviously red-shifted in Figure 6 and 7. With the modified electronic structures of RS models in the section S2, the spectra obtained via BSE show the apparent blue-shift in Figure S3.

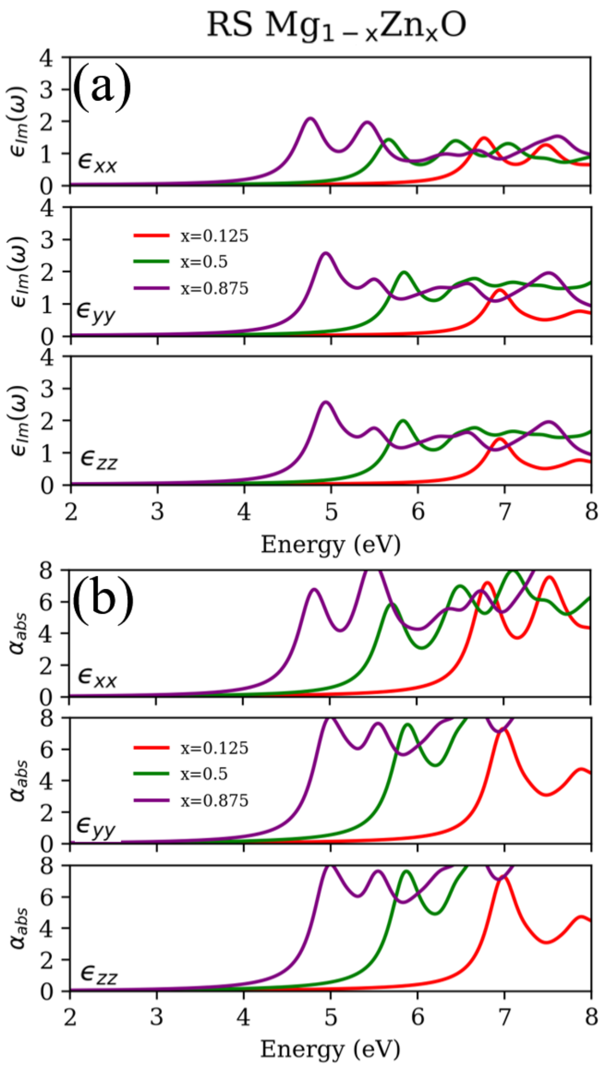

Figure S3. (a) Imaginary part of the dielectric function (ϵ_{Im}) and (b) absorption coefficient (α_{abs}) for rocksalt (RS) $Mg_{1-x}Zn_xO$ based on Bethe-Salpeter equation with the modified electronic structures in the section S2. To simplify the expressions, the selected concentration of x = 0.125 (red), 0.5 (green) and 0.875 (purple) are presented to demonstrate the blue-shifted phenomenon.

References

1. Ma, X.; Wu, Y.; Lv, Y.; Zhu, Y. Correlation Effects on Lattice Relaxation and Electronic Structure of ZnO within the GGA+U Formalism. *J. Phys. Chem. C* **2013**, *117*, 26029–26039. doi:10.1021/jp407281x.
